# Ectopic recurrence craniopharyngioma: series report and literature review

**DOI:** 10.1186/s41016-023-00326-3

**Published:** 2023-05-06

**Authors:** Chenxing Ji, Haixia Cheng, Xiang Zhou, Xiaoyun Cao, Nidan Qiao, Chengzhang Shi, Yichao Zhang, Zhao Ye, Yao Zhao

**Affiliations:** 1 Department of Neurosurgery, National Center for Neurological Disorders, Huashan Hospital, Shanghai Medical College, Fudan University, Shanghai, 200040 China; 2 Neurosurgical Institute of Fudan University, Shanghai, 200040 China; 3 Department of Pathology, Huashan Hospital, Shanghai Medical College, Fudan University, Shanghai, 200040 China; 4 Shanghai Key Laboratory of Brain Function Restoration and Neural Regeneration, Shanghai, 200040 China; 5 State Key Laboratory of Medical Neurobiology and MOE Frontiers Center for Brain Science, Institutes of Brain Science, Fudan University, Shanghai, China; 6 National Clinical Research Center for Aging and Medicine, Huashan Hospital, Fudan University, Shanghai, 200040 China

**Keywords:** Ectopic recurrence, Craniopharyngioma, Case report, Literature review

## Abstract

**Background:**

Craniopharyngioma is a common intracranial tumor located in the sellar-suprasellar region. Due to the involvement of adjacent structures, it can lead to increased intracranial pressure, visual impairment, and endocrine deficiencies. Surgical resection is the primary treatment, but it is a tough challenge to achieve total resection, which will led to the frequency of recurrences and progressions. Among them, distant spread is extremely rare, but important complication, identifying and providing proper therapy, is crucial.

**Methods:**

We report two cases of ectopic recurrence craniopharyngioma and make a literature review for the published similar case reports.

**Results:**

Our literature review revealed 63 cases (including our patient). The onset age in children group and adult group ranges from 2–14 years old (6.70 ± 3.33) to 17–73 years old (40.63 ± 15.58), while the interval year between tumor initiation and ectopic recurrence ranges from 0.17–20 (7.28 ± 6.76) years to 0.3–34 (6.85 ± 7.29). Achieving gross total resection seems not to prevent the ectopic recurrence. The major pathology of ectopic recurrence craniopharyngioma is adamantinomatous type. The most common site of ectopic recurrence is frontal lobe. According to the pathogenesis, 35 cases were seeding along the surgical approach, and 28 cases were seeding via the CSF pathway.

**Conclusion:**

Ectopic recurrence craniopharyngioma is rare, but it can lead to serious symptoms. Delicate surgical procedure can help to reduce the risk of ectopic recurrence, and standardized follow-up can provide valuable information for treatment.

## Background

Craniopharyngioma is a rare malformational tumor of low histological malignancy, and two primary subtypes have been recognized (adamantinomatous and papillary) as yet. It originates from the remnants of Rathke’s pouch and mainly occurs in sellar-suprasellar region [1]. However, some rarely distant spread had been reported. This ectopic recurrence mainly disseminates along the surgical approach or via the cerebrospinal fluid (CSF) pathway. In this study, we report two cases of ectopic recurrence craniopharyngioma and make a literature review for the published similar case reports to introduce some experience with the management of ectopic recurrence craniopharyngioma. All the patients consented to the procedure.

## Case presentation

### Case 1

**Fig. 1:**
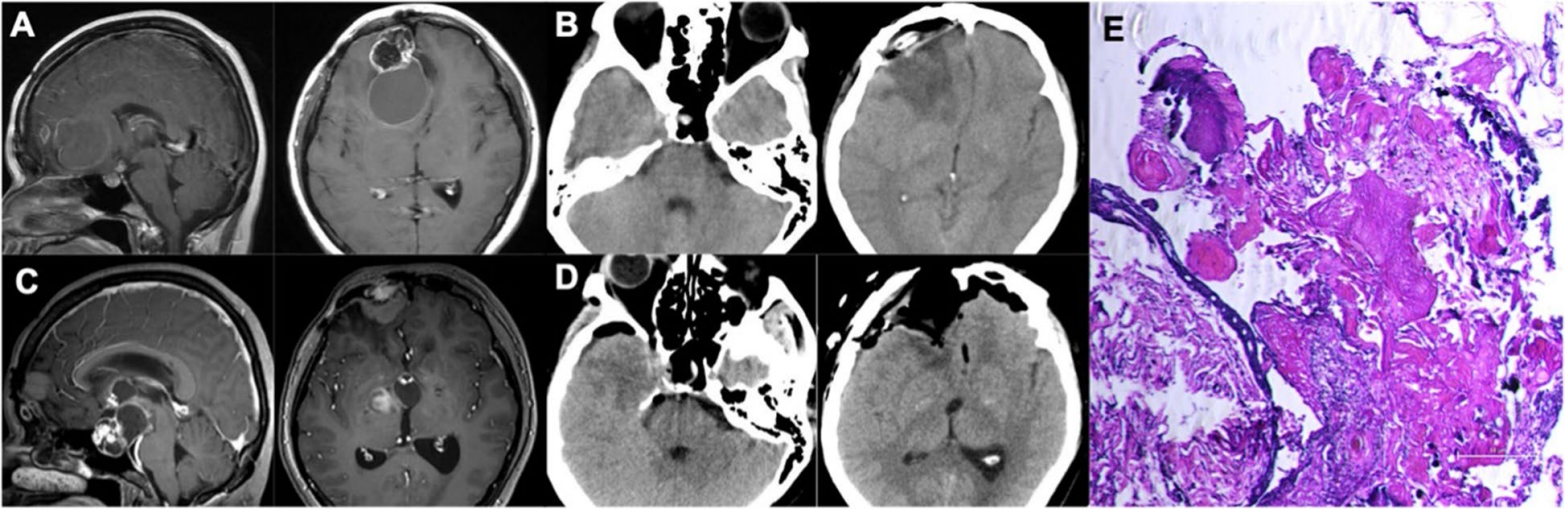
MRI, CT scans, and pathological features of case 1.** A** Preoperative MRI showed a cystic solid lesion in the sellar-suprasellar region extending to the right frontal lobe in 2012. **B** Postoperative CT scan showed total resection of the right frontal lesion. **C** MRI scan showed tumor recurrence in both the sellar-suprasellar region and the right frontal lobe in 2021. **D** Postoperative CT scan showed total resection of the two lesions. **E** The pathological features showed typical morphology of adamantinomatous craniopharyngioma

A 49-year-old female patient initially complained of vision loss and nausea in 2005. A sellar lesion had been detected and subtotally resected by craniotomy. The lesion was histologically determined to be an adamantinomatous craniopharyngioma. As supplementary therapy, the patient received gamma-knife treatment in 3 months and 12 months after operation. In 2012, the patient suffered severe nausea and vomiting again. The MR scan displayed tumor recurrence in the sellar region, with a cystic lesion extend to the right frontal lobe (Fig. 1A). These two lesions had been simultaneously resected through a transcranial surgery (Fig. 1B), and the pathology showed both were adamantinomatous craniopharyngioma (*Ki-67* = 3%). However, in 2021, it had been found tumor recurrence in the sellar region; even worse, there was a new isolated lesion in the right frontal lobe (Fig. 1C). She received tumor resection therapy via the same transcranial approach (Fig. 1D), and the pathological staining confirmed the adamantinomatous craniopharyngioma with Ki-67 2% (Fig. 1E).

### Case 2

**Fig. 2:**
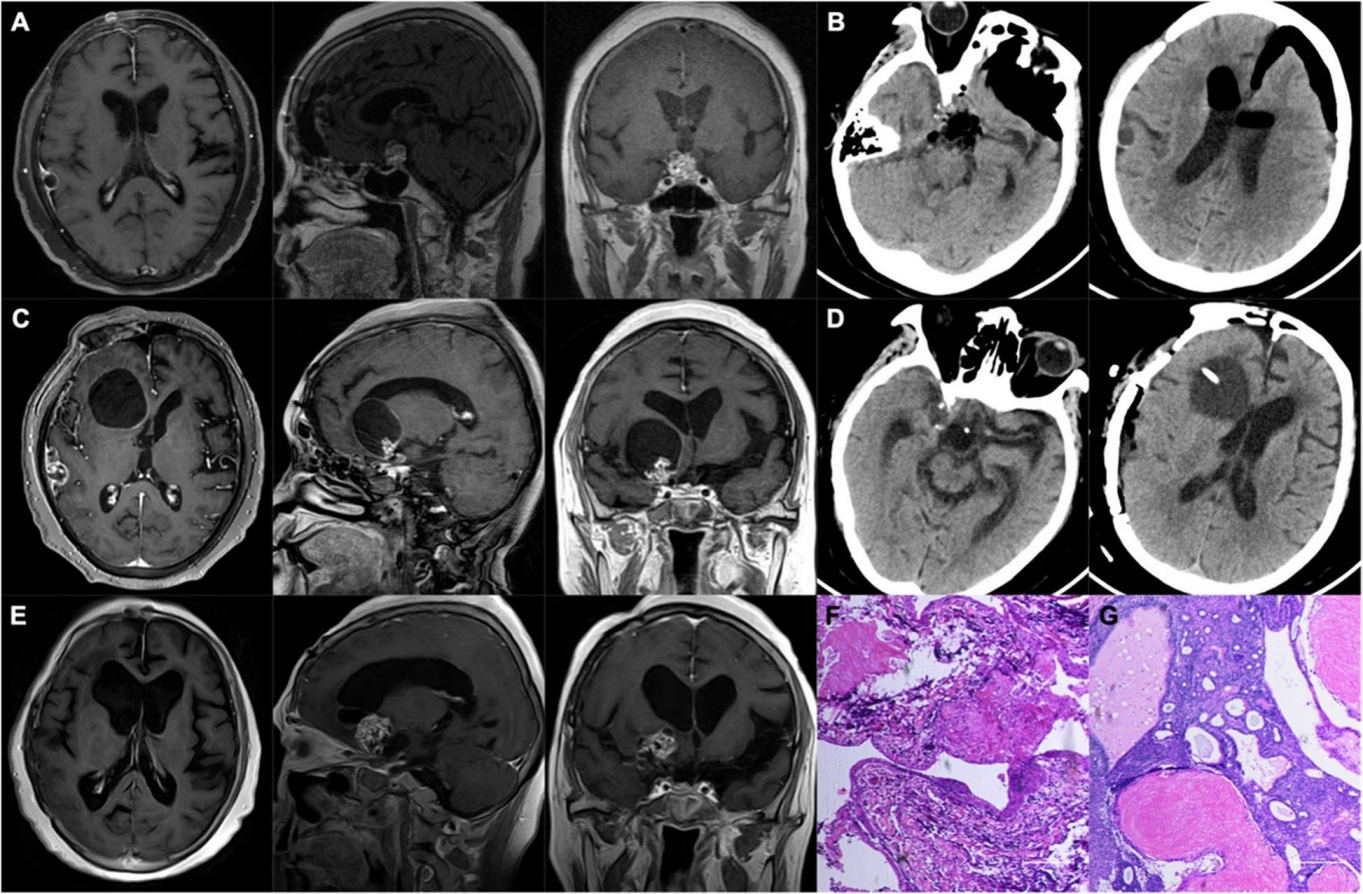
MRI, CT scans, and pathological features of case 2.** A** Preoperative MRI showed a cystic and solid lesion in the sellar-suprasellar region and a small lesion in the right temporal lobe in 2019. **B** CT scan after endoscopic transsphenoidal surgery. **C** MRI scan showed tumor recurrence with cyst in the suprasellar region and enlargement of the lesion in the right temporal lobe in 2020. **D** CT scan after transcranial surgery and Ommaya reservoir implantation. **E** MRI scan showed tumor recurrence again in the suprasellar region in 2021. **F** The pathological features of suprasellar lesion showed adamantinomatous craniopharyngioma. **G** The pathological features of right temporal lobe lesion also showed adamantinomatous craniopharyngioma

A 63-year-old female patient suffered from bilateral vision loss for 6 months before the first diagnosis as craniopharyngioma in 2017. The patient received a subtotal resection of the tumor by craniotomy in other hospital. Her vision had partially recovered after operation. However, in 2019, the patient felt vision loss again, while the MR scan showed the tumor had recurred not only in the initial site but also in the right temporal lobe with a 1.2 × 0.9-cm lesion (Fig. 2A). Due to the small lesion volume and lack of related symptoms, this patient decided to resect the tumor in the suprasellar region by transsphenoidal surgery firstly (Fig. 2B) and did a close follow-up to the right temporal lesion. One year postoperatively in 2020, the patient suffered serious symptoms like worse vision, bluntness, and drowsiness. The MR scan displayed tumor recurrence in the suprasellar region, and the right temporal lesion had enlarged (Fig. 2C). A craniotomy surgery was performed to resect both lesions and place an Ommaya catheter in the cyst (Fig. 2D). In 2021, unfortunately, the tumor relapsed rapidly (Fig. 1E). The pathology of both lesions was adamantinomatous craniopharyngioma (Fig. 2 F–G), with a higher Ki-67 index (5%) of the ectopic recurrence compared with that (3%) in initial site.

## Discussion

Craniopharyngioma is a benign epithelial tumor and accounts for 0.5–2.5 cases per 1 million population every year [2–4]. It originates from the sellar region, specifically the craniopharyngeal duct [1]. The main symptoms are caused by compression of adjacent tissues such as visual disturbance (optic nerve), endocrine disorder (pituitary gland), personality changes (hypothalamus), and hydrocephalus (third ventricle) [5, 6]. The primary therapy is surgical resection. However, due to the unpredictable growth pattern and involvement of critical anatomical structures, it is difficult to achieve total resection and apt to relapse.

Ectopic recurrence craniopharyngioma is rare. Up to date, only 61 patients with 63 cases had been described in the literature including our own [7–53] (Table 1). All the cases are exhibited by case reports; thus, it is difficult to make epidemiology statistics. According to previous studies listed in Table 1, the incidence of ectopic recurrence craniopharyngioma has been 0 ~ 4.7% and accounts for 7 ~ 20% of all recurrences approximately. As craniopharyngioma has a bimodal age distribution, with peak incidence rates observed in children aged 5–14 years and adult aged 50–74 years [54], the incidence of ectopic recurrence between children group and adult group has no significant difference based on our statistic. The onset age in children group and adult group ranges from 2–14 years old (6.70 ± 3.33) to 17–73 years old (40.63 ± 15.58), while the interval year between tumor initiation and ectopic recurrence ranges from 0.17–20 (7.28 ± 6.76) years to 0.3–34 (6.85 ± 7.29), respectively. There is no significant gender difference on ectopic recurrence. By reviewing the treatment history, achieving gross total resection seems not to prevent the ectopic recurrence during first operation. What’s more, 16 patients who received postoperative adjuvant radiotherapy still suffer from the ectopic recurrence. There is no accurate data about the long-term prognosis of ectopic recurrence due to the scarcity of clinical cases. According to current data, no recurrence was observed at a longest follow-up duration of 12 years [26]. But one patient died due to local recurrence in sellar region [35].

**Table 1:** The characteristics of ectopic recurrence craniopharyngioma

Researcher	Age (year)	Gender	Initial pathology	Previous treatment	Ectopic recurrence location	Tumor size (cm)	Ectopic recurrence pathology	Ectopic recurrence mechanism	Interval after initial (year)
Present study	63	Female	ACP	1st cranio STR 2nd TSS STR	R temporal lobe	2.6 × 1.3	ACP (Ki-67 = 5%)	CSF	3
	49	Female	ACP	1st cranio STR + GK 2nd cranio GTR	R frontal lobe	1st 5.7 × 4.3; 2nd 3.4 × 2.1	ACP (1st KI-67 = 3% 2nd Ki-67 = 2%)	Surgical approach	1st 7 2nd 16
Cai (2019) [7]	28	Male	ACP (Ki-67 = 10%)	Cranio GTR	R temporal lobe	6 × 4 × 5	ACP (Ki-67 = 20%)	CSF	1
Renfrow (2018) [8]	14	Female	ACP	1st cranio GTR 2nd cranio GTR 3rd RT	L lateral ventricle	1.2	ACP	CSF	12
Mahdi (2018) [9]	24	Male	ACP	Cranio GTR	R CPA	Unclear	ACP (KI-67 = 5%)	CSF	1
Jian (2017) [10]	42	Male	PCP	Cranio GTR	R frontal lobe	5 × 4	PCP (Ki-67 = 3%)	CSF	0.3
Carleton-Bland (2017) [11]	10	Male	ACP	1st cranio STR 2nd cranio STR + RT	R lateral ventricle	2 × 1.8	ACP	CSF	2
Du (2017) [12]	6	Female	ACP	Cranio GTR	R frontal lobe	6.5 × 5 × 6	ACP (Ki-67 = 1%)	Surgical approach	5
	4	Male	ACP	Cranio GTR	Fourth ventricle	Unclear	ACP (Ki-67 = 1%)	CSF	4
Clark (2015) [13]	33	Female	ACP	1st cranio GTR 2nd cranio GTR	L Sylvian fissure	3.2 × 2.2	ACP	Surgical approach	34
Yang (2015) [14]	35	Male	Unclear	Cranio GTR	R frontal lobe	6 × 5.5 × 5	PCP (Ki-67 = 1%)	Surgical approach	9
	46	Male	PCP (Ki-67 = 2%)	Cranio GTR	Interhemisphere	2.2 × 1.7 × 1.4	PCP (Ki-67 = 5%)	Surgical approach	2
	42	Male	ACP	1st cranio GTR 2nd cranio GTR	R frontal lobe	5.5 × 5 × 5	ACP (Ki-67 = 1%)	Surgical approach	6
Gonçalves (2014) [15]	49	Male	ACP	Cranio GTR	R frontal lobe	3	ACP	Surgical approach	5
Jakobs (2012) [16]	61	Female	ACP	1st cranio GTR 2nd RT	R frontal bone	1.9 × 1.8	ACP (Ki-67 = 5%)	Surgical approach	11
Elfving (2011) [17]	4	Female	ACP (KI-67 < 1%)	1st cranio GTR 2nd cranio STR + RT	R frontal lobe	Unclear	ACP (KI-67 = focal 15%)	Surgical approach	11
Salunke (2011) [18]	5	Female	Unclear	Cranio STR	R Sylvian fissure	Unclear	ACP	Surgical approach	0.92
de Blank (2011) [19]	5	Female	Unclear	1st cranio GTR 2nd RT	L CPA	4.5 × 4.1	Unclear	Surgical approach	17
Kordes (2011) [20]	7	Male	ACP	Cranio PR + TSS + RT	R parietal lobe	2	ACP	Surgical approach	1.25
Wang (2010) [21]	3	Male	ACP	Cranio GTR + RT	R frontal lobe	Unclear	ACP	Surgical approach	2
Lermen (2010) [22]	45	Male	ACP	1st cranio STR 2nd cranio GTR	Lumber space	Unclear	ACP	CSF	0.5
Schmalisch 2010 [23]	11	Male	ACP (KI-67 = 1%)	Cranio GTR	R Sylvian fissure	Unclear	ACP (KI-67 = 2%)	Surgical approach	2
	23	Female	ACP (KI-67 = 8%)	1st cranio STR 2nd TSS	R frontal lobe	Unclear	ACP (KI-67 = 8%)	Surgical approach	4
	32	Male	ACP (KI-67 = 2%)	Cranio GTR	R parietal lobe	Unclear	ACP (KI-67 = 5%)	CSF	10
Romani (2010) [24]	18	Female	ACP	Cranio GTR	Interhemisphere	Unclear	ACP	Surgical approach	4
Frangou (2009) [25]	10	Male	ACP	1st cranio STR 2nd cranio STR 3rd cranio STR + RT	R parietal lobe	Unclear	ACP	CSF	4
Elliott (2009) [26]	3	Female	ACP	Cranio GTR	Prepontine cistern	0.5	ACP	CSF	10
	2	Male	ACP	1st cranio STR 2nd cranio GTR 3rd cranio GTR	L CPA	1.5	ACP	CSF	3.5
	3	Female	ACP	Cranio GTR	R Sylvian fissure	2	ACP	Surgical approach	1.67
	6	Male	ACP	1st cranio STR 2nd cranio GTR	L frontal lobe	1	ACP	CSF	7.08
Bikmaz (2009) [27]	37	Female	Unclear	Unclear	Prepontine	Unclear	ACP	CSF	15
	32	Male	Unclear	GTR	R frontal lobe	Unclear	Non-ACP	Surgical approach	9
	12	Male	Unclear	STR + RT	B CPA	Unclear	Unclear	CSF	12
Novák (2008) [28]	48	Male	Unclear	Unclear	Posterior fossa	Unclear	Unclear	CSF	19
Jeong (2006) [29]	8	Female	ACP (KI-67 = 3%)	Cranio GTR	R frontal lobe	Unclear	ACP (KI-67 = 3%)	Surgical approach	4
Yamada (2006) [31]	17	Female	ACP (KI-67 = 4.2%)	Cranio STR + RT	L frontal lobe	Unclear	ACP (KI-67 = 7.4%)	CSF	5
Bianco (2005) [31]	27	Female	ACP	1st cranio STR 2nd cranio GTR	L temporal lobe cortex	1	ACP	Surgical approach	10
Kawaguchi (2005) [32]	50	Female	Unclear	1st unclear 2nd cranio GTR	L frontal lobe	Unclear	ACP	Surgical approach	2
Ishiia (2004) [33]	2	Male	Unclear	1st cranio STR 2nd cranio GTR	R frontal lobe	Unclear	ACP	Surgical approach	0.17
Liu (2002) [34]	65	Female	Unclear	Cranio GTR	R frontal lobe	4.5 × 6.2 × 3	ACP	Surgical approach	3
Nomura (2002) [35]	17	Female	ACP (KI-67 = 3%)	1st cranio STR 2nd cranio STR + RT	R frontal and temporal lobe	Unclear	ACP (F MIB = 6.2%, T MIB = 5.1%)	CSF	3.92
Fuentes (2002) [36]	32	Male	ACP	GTR	R frontal lobe	Unclear	ACP	Surgical approach	5
	11	Male	ACP	GTR	Unclear	Unclear	ACP	Surgical approach	3
	9	Male	ACP	GTR	R frontal lobe R temporal lobe	Unclear	ACP	Surgical approach	10
Elmaci (2002) [37]	62	Female	PCP (KI-67 = 1%)	Cranio GTR	L temporal lobe	4 × 3 × 2.5	PCP (KI-67 = 3%)	CSF	2
Novegno (2002) [38]	6	Male	ACP	Cranio GTR	1st L frontal lobe 2nd L pontine R cerebral basal	Unclear	Unclear	1st surgical approach 2nd CSF	1st 3 2nd 4
Lee (2001) [39]	26	Male	PCP	Cranio STR + GK	Lumber space	Unclear	PCP	CSF	1.58
Ito (2001) [40]	62	Male	ACP	Cranio GTR	R frontal lobe	Unclear	ACP	CSF	3
Freitag (2001) [41]	61	Female	Unclear	Unclear	R frontal lobe	Unclear	Unclear	Surgical approach	5
Kim (2001) [42]	Child	Unclear	Unclear	Unclear	Internal auditory canal	Unclear	Unclear	CSF	Unclear
Gupta (1999) [43]	73	Male	ACP	Cranio GTR	L parietal lobe and L frontal lobe	2	ACP	CSF	7
Lee (1999) [44]	31	Male	Unclear	Cranio GTR	R frontal lobe	1	Unclear	Surgical approach	5
Israel (1995) [45]	12	Male	Unclear	Cranio GTR	R frontal lobe	4	Unclear	Surgical approach	2
Keohane (1994) [46]	7	Female	Unclear	Cranio STR + RT	L CPA	Unclear	ACP	CSF	26
Tomita (1993) [47]	Child	Unclear	Unclear	Unclear	R frontal lobe	Unclear	Unclear	Surgical approach	Unclear
Malik (1992) [48]	6	Male	ACP	1st cranio PR + RT 2nd cranio PR 3rd cranio GTR	R frontal lobe	3.5	ACP	Surgical approach	21
Tomita (1992) [49]	23	Female	Unclear	1st cranio PR 2nd RT	R CPA, interpeduncle, prepontine	Unclear	Unclear	CSF	25
Gökalp (1991) [50]	3	Male	Unclear	Cranio GTR	Fourth ventricle	3 × 3 × 3	Unclear	CSF	20
Ragoowansi (1991) [51]	47	Male	Unclear	1st stereotactic biopsy 2nd cranio GTR	R Sylvian fissure	2.5 × 1.5	Unclear	Surgical approach	1
Barloon (1988) [52]	5	Male	Unclear	1st cranio STR RT and cyst aspiration	R frontal lobe	Unclear	Unclear	Surgical approach	5
Baba (1978) [53]	7	Female	Unclear	Cranio STR	Prepontine C3	Unclear	Unclear	CSF	7

The diameter of ectopic recurrence tumor ranges from 0.5 to 6.5 (3.14 ± 1.73) cm. The pathological results of ectopic recurrence craniopharyngioma demonstrate that the adamantinomatous type constituted the majority with the number of 36, while the papillary type accounted for only 4 cases and 20 cases’ pathology was unclear. The Ki-67 index of ectopic tumor does not show a remarkable increasement than that of initial tumor. The most common site of ectopic recurrence is frontal lobe, followed by cerebellopontine angle (CPA), temporal lobe, parietal lobe, and others. Currently, there are two views on the mechanism about ectopic recurrence, along the surgical approach and the CSF pathway. Transcranial surgery for craniopharyngioma via pterion is the most frequently used approach that can achieve sufficient surgical field and better protection of nerves and vessels. But it is believed that the tumor cells would disseminate or seed during the operation, and that is the reason why frontal lobe was the most common site of ectopic recurrence, so did the recurrence in Sylvian fissure. The ectopic recurrence mechanism of case 1 in our study belonged to this type. Ectopic recurrence in CPA, temporal and parietal lobe, and lumber space, which far from the surgical route, were considered as the dissemination of tumor cells via CSF pathway. That is the mechanism of case 2; the recurrence site in temporal lobe was far away from the primary surgical area. After statistical analysis, 35 ectopic recurrence cases were due to the seeding along the surgical approach, and 28 cases were due to the seeding via the CSF pathway.

Ectopic recurrence craniopharyngioma is rare, but it can lead to serious symptoms according the location such as hearing loss, disequilibrium, epilepsy, behavioral, and personality change. Thus, some delicate surgical procedure such as strictly separating the tumor by cottonoids, carefully aspirating the cystic fluid to avoid the contamination of CSF, and adequate irrigation in surgical field can help to reduce the risk of ectopic recurrence, and standardized follow-up can provide valuable information for treatment.

## Data Availability

Not applicable.
